# Chromatophore Activity during Natural Pattern Expression by the Squid *Sepioteuthis lessoniana*: Contributions of Miniature Oscillation

**DOI:** 10.1371/journal.pone.0018244

**Published:** 2011-04-01

**Authors:** Mamiko Suzuki, Tetsuya Kimura, Hiroto Ogawa, Kohji Hotta, Kotaro Oka

**Affiliations:** 1 Graduate School of Science and Technology, Keio University, Yokohama, Kanagawa, Japan; 2 Laboratory for Alzheimer Disease, RIKEN Brain Science Institute, Wako, Saitama, Japan; 3 Graduate School of Life Science, Hokkaido University, Sapporo, Hokkaido, Japan; University of Arizona, United States of America

## Abstract

Squid can rapidly change the chromatic patterns on their body. The patterns are created by the expansion and retraction of chromatophores. The chromatophore consists of a central pigment-containing cell surrounded by radial muscles that are controlled by motor neurons located in the central nervous system (CNS). In this study we used semi-intact squid (*Sepioteuthis lessoniana*) displaying centrally controlled natural patterns to analyze spatial and temporal activities of chromatophores located on the dorsal mantle skin. We found that chromatophores oscillated with miniature expansions/retractions at various frequencies, even when the chromatic patterns appear macroscopically stable. The frequencies of this miniature oscillation differed between “feature” and “background” areas of chromatic patterns. Higher frequencies occurred in feature areas, whereas lower frequencies were detected in background areas. We also observed synchronization of the oscillation during chromatic pattern expression. The expansion size of chromatophores oscillating at high frequency correlated with the number of synchronized chromatophores but not the oscillation frequency. Miniature oscillations were not observed in denervated chromatophores. These results suggest that miniature oscillations of chromatophores are driven by motor neuronal activities in the CNS and that frequency and synchrony of this oscillation determine the chromatic pattern and the expansion size, respectively.

## Introduction

Unshelled cephalopods have the extraordinary ability to change their body color pattern in less than a second for camouflage and intraspecies communication, and the color patterning is often sustained for seconds or minutes [1]. The patterns are mediated by retraction and expansion of chromatophore organs (referred to hereafter as chromatophore(s)). A chromatophore consists of a central pigment-containing cell surrounded by 19–27 radial muscles that are controlled by motor neurons in the brain [2], [3], [4], [5]. Contraction of the radial muscles causes an expansion of the pigment-containing cell.

In isolated skin preparations or in semi-intact preparations that contain the mantle and the brain, electrical stimulation of a chromatophore motor nerve induces twitch-like contractions of the radial muscles in several chromatophores [6], [7], [8]. Increasing the frequency of the stimulating pulse causes summation of the contractions and results in “flickering” behavior, which is produced by rapid mini-contraction/relaxation cycles of the chromatophore muscles. Chromatophores can exhibit not only full expansion but also intermediate expansion in which the muscles produce graded contractions. These variations in the state of expansion (rather than intensity of pigment) of dark chromatophores are responsible for variations in contrast of the chromatic patterns produced [9], [10], [11], [12]. In intact cuttlefish, recordings of the electrical activity from motor nerves innervating chromatophores in black spot areas of a deimatic chromatic pattern showed an increased firing rate when the pattern was displayed [13]. Thus, firing frequencies in motor neurons control the chromatic pattern.

Electrical stimulus studies have been used to examine what causes the chromatophore to expand, but little is known about how chromatophores actually behave in intact squid, especially during chromatic pattern expression. In the present study, we analyzed behavior of individual black chromatophores in semi-intact specimens of the squid *Sepioteuthis lessoniana*. We also compared chromatophore behavior between “feature” and “background” areas.

## Results

### Spontaneous chromatophore behavior and chromatic patterns in semi-intact squid

**Figure 1:**
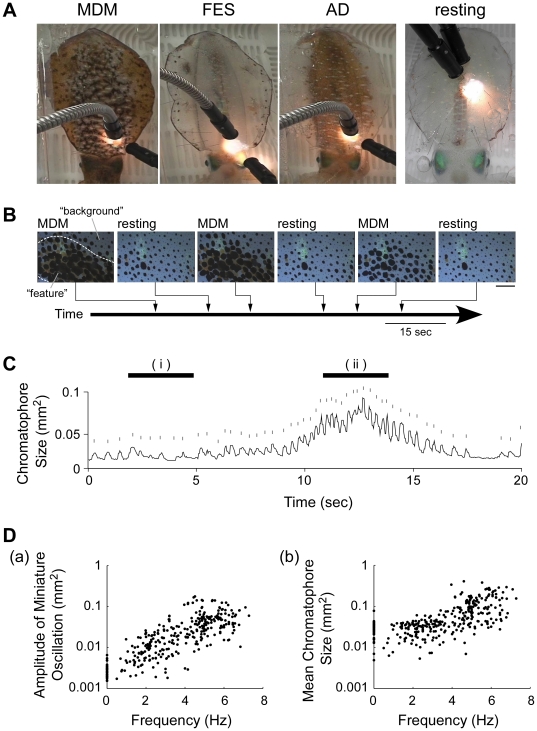
The expression of chromatic patterns and chromatophore behavior in semi-intact *Sepioteuthis lessoniana*. A: Three chromatic patterns observed in this study: mottled dorsal mantle (MDM), fin edge spots (FES), and all dark (AD). Body color in the resting state is clear (resting). B: A typical time course of MDM pattern expressions. The pictures show a part of the dorsal mantle skin in which the MDM pattern appeared at different intensities and then disappeared (resting). The appearance time of each picture is indicated in the trace. The broken lines in the left picture indicate the boundary between feature and background areas. Scale bar: 1.5 mm. C: A temporal size profile of an individual chromatophore. Upper short lines above the each trace show the contribution of miniature oscillation to the expansions. Top lines indicate the 3.3 s periods during which the oscillation frequencies for smaller chromatophore size (i) and larger size (ii) were calculated. D: Relationship between oscillation frequency and amplitude of miniature oscillation (a) and between oscillation frequency and mean chromatophore size (b) during nine periods of 3.3 s. The amplitude of miniature oscillation indicates the maximum amplitude value in the expansion and retraction in each frame (See figure S1). The mean chromatophore size is the average value during the 3.3 s. A logarithmic scale was used on the Y-axis. Data were analyzed from 336 individual black chromatophores on a total of six areas from three animals.

We recorded three chromatic patterns – mottled dorsal mantle (MDM), fin edge spots (FES), and all dark (AD) (Fig. 1A) – spontaneously displayed by three un-anesthetized squid loosely fixed in a container. In the MDM pattern (a camouflage pattern), groups of expanded chromatophores on the dorsal mantle surface created the dark mottled feature on a pale background of unexpanded chromatophores. In the FES pattern, several dark spots consisting of expanded chromatophores appeared along the edge of the fin. In the AD pattern, almost all of the chromatophores on the body surface were expanded (Fig. 1A). From the three squid, we observed 25 MDM, 8 FES, and 21 AD pattern expressions during the total observation period of 98 min. The series of video frames shows that the feature (e.g., MDM) was expressed repeatedly in the same area (Fig. 1B).

All expanded chromatophores showed oscillatory activities with repetitive miniature expansions and retractions in comparison with macroscopic changes in the chromatophore size during chromatic pattern formation (Fig. 1C). The miniature oscillations were characterized by rapid small expansions that usually occurred in a single frame interval (<33 ms) and relatively slow retractions that required over two frames (>66 ms) to reach the bottom state of the oscillation (Fig. S1). Upper short lines above the trace in Figure 1C indicate the miniature expansions. Intervals between the miniature expansions became shorter with increasing chromatophore size (i.e., oscillation frequency increased). We termed the macroscopic change in the chromatophore size, which was the low-frequency component in the chromatophore behavior, as *basal expansion* to distinguish it from the miniature oscillatory activity, which was the high-frequency component. The oscillation frequency was estimated to be 2.1 Hz at the smaller size of the basal expansion (Fig. 1Ci) and 4.0 Hz at the larger size (Fig. 1Cii). Further analysis of temporal size change was performed for short time periods (3.3 s) when the squid exhibited relatively stable chromatic patterns (total of 9 periods, 239 different chromatophores, 3 squid). The frequency and amplitude of the miniature oscillations were determined from the high-frequency component estimated by differentiating the temporal profile of the chromatophore size (Fig. S1), and the basal expansion size was taken as the mean chromatophore size during the measurement period of 3.3 s. This analysis indicated that increasing the oscillation frequency resulted in larger oscillation amplitude, which was defined as the difference between the maximum and minimum values of each differentiated profile of the chromatophore size (Fig. 1Da). However, we also found that the mean chromatophore size during the 3.3 s time periods (i.e., basal expansion size) was non-linearly related to its oscillation frequency, with an inflection point near 4 Hz (Fig. 1Db). This result suggests that all well-expanded chromatophores undergo miniature oscillations at frequencies >4 Hz.

**Figure 2:**
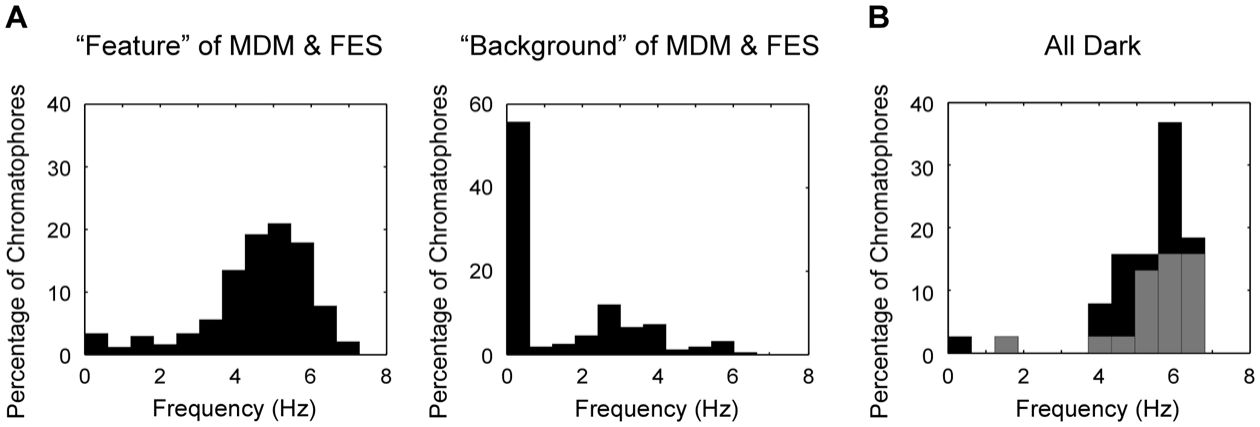
Oscillation frequencies of chromatophores. A: Frequency histograms show the distribution of oscillation frequency in the feature areas (n = 230; 111 different chromatophores, 2 animals) and background areas (n = 147; 68 different chromatophores, 2 animals) for the MDM and FES patterns. B: Frequency distribution during the AD pattern for 38 of the same chromatophores represented in Fig. 2A (see text). The gray area indicates the percentage of chromatophores located in the MDM feature area, and the black area indicates chromatophores located in the background area.

We compared the oscillation frequency of chromatophores in the feature areas (dark areas in the MDM or FES patterns) with those in the background areas for 3.3 s during expression of chromatic patterns (Fig. 2A). The frequency distribution of feature area chromatophores showed a skewed distribution, with one peak at 4.9–5.3 Hz and an average oscillation frequency of 4.7 Hz (sample variance, *s* ^2^ = 1.7, n = 218) (Fig. 2A). In background areas, many chromatophores were at rest, and expanded chromatophores oscillated at a lower frequency (average 3.2 Hz, *s* ^2^ = 1.4, n = 64) (Fig. 2A). Figure 2B shows the oscillation frequency during AD expression of some of the same chromatophores that had been measured during MDM expression (n = 20 from feature areas, n = 18 from background areas). Their oscillation frequency exhibited a similar distribution to that of the feature area observed for the MDM pattern: mean 5.4 Hz (*s* ^2^ = 1.0, n = 37). The chromatophores on the background area of the MDM that oscillated at lower frequencies also oscillated at higher frequencies in the AD condition.

**Figure 3:**
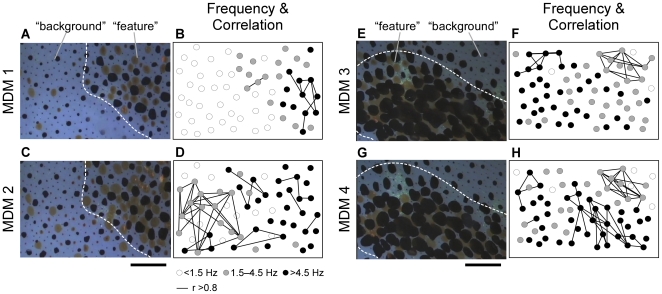
Spatial correlation maps of miniature oscillation during four chromatic pattern expressions. Pictures on the left of each panel (A, C, E, and G) are parts of different MDM expressions. Movies of MDMs 1 and 2 and MDMs 3 and 4 were recorded in the same areas individually. Broken lines indicate the border between feature and background areas. Scale bar: 1.5 mm. Pictures on the right of each panel (B, D, F, and H) show the oscillation frequency in individual chromatophores and oscillatory synchrony among individual chromatophores in that expression. The circles indicate each chromatophore and the color of each circle indicates its oscillation frequency: white is <1.5 Hz, gray is 1.5–4.5 Hz, and black is >4.5 Hz. The pair of chromatophores connected by a line corresponds to highly correlated oscillatory behavior (correlation coefficient, r>0.8). (See video S1 for the expressed MDM 1 and video S2 for the expressed MDM 2.).

**Figure 4:**
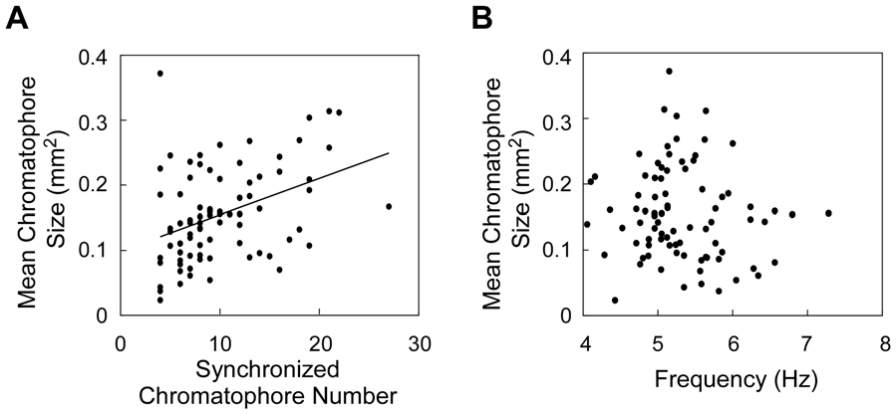
Relationship between mean chromatophore size and synchronized chromatophore number (A) and between mean chromatophore size and oscillation frequency (B) in chromatophores oscillating above 4 Hz represented in **Fig. 3** (83 chromatophores). A: The synchronized chromatophore number indicates the number of chromatophores with highly correlated oscillatory behavior (correlation coefficient, r>0.6). There is a correlation between the mean chromatophore size and synchronized chromatophore number, r = 0.40 (P<0.001). The line in the figure represents the best-fitting line. B: The mean chromatophore size shows no correlation with the oscillation frequency, r = 0.11 (P = 0.34).

Figure 3 shows four examples of the results of our analysis of synchrony among simultaneous recorded miniature oscillations. This analysis revealed the following tendencies: (1) Synchronization occurred among chromatophores oscillating at frequencies >1.5 Hz (see chromatophores indicated by while circles); (2) synchronization among the miniature oscillations was detectable in relatively distant chromatophores (see distribution of chromatophores connected by a line); (3) different synchronized groups were simultaneously observed (see connected chromatophores in Fig. 3B, D, F, and H); (4) each group size was varied (see numbers of connected chromatophores); (5) synchronized groups were not fixed (see Fig. 3B vs. D and F vs. H); and (6) synchronization occurred in both feature and background areas; and (7) synchronized groups did not cross the border between the feature and background areas. These observations demonstrated that assembly behavior of chromatophores occurred in a chromatic pattern-dependent manner. To examine the relationship between basal expansion size and synchrony of the oscillatory behavior in chromatophores oscillating over 4 Hz, the mean chromatophore sizes during the measurement period of 3.3 s were plotted against the number of chromatophores synchronized with more than 3 chromatophores (correlation coefficient, r>0.6). There was a positive linear correlation between the mean chromatophore size and the number of synchronized chromatophores (correlation coefficient, r = 0.40) (Fig. 4A). On the other hand, the mean chromatophore size did not correlated with the oscillation frequency as shown in Fig. 1Db (correlation coefficient, r = 0.11) (Fig. 4B). These results suggest that the basal expansion size is defined by the synchronized chromatophore number, not the oscillation frequency (Fig. 4). The oscillatory system produces two kinds of motor output, chromatic pattern formation and expansion size of individual chromatophores, by controlling frequency and synchronization of the oscillatory behavior.

### Chromatophore behavior controlled by the brain

**Figure 5:**
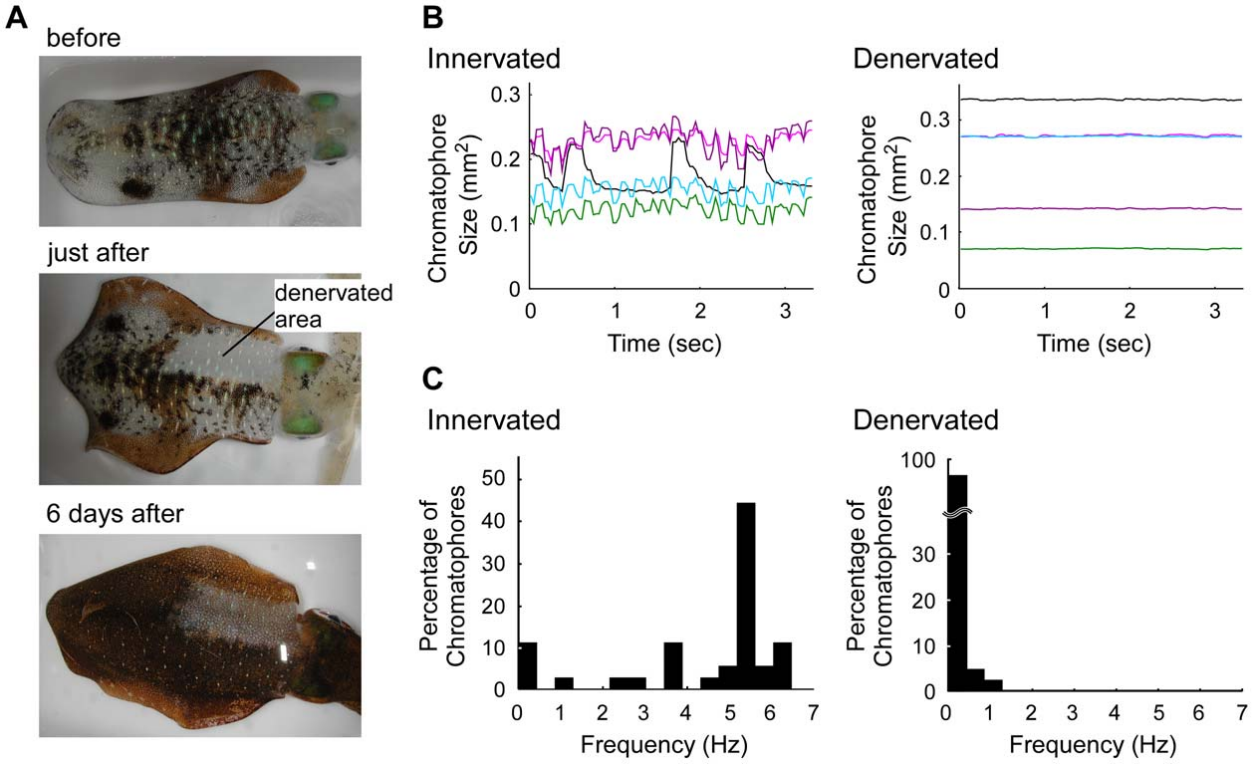
Behavior of chromatophores on innervated and denervated regions in semi-intact squid. A: Images of dorsal mantle including normal and denervated regions before the operation (upper), immediately after recovery from the operation (middle), and 6 days later (lower). B: Temporal profile of expanded sizes of chromatophores in innervated and denervated regions 6 days after the operation. Each line represents individual chromatophores. C: Distribution of oscillation frequencies of chromatophores in the innervated area (36 chromatophores) and denervated area (43 chromatophores).

Expanded chromatophores repeat a rapid expansion-retraction cycle at various frequencies (Fig. 1). To determine whether these miniature oscillatory activities are regulated by the motor neurons in the central nervous system (CNS), we interrupted efferent control by cutting the pallial nerve on one side of the body. Eighteen squid were used for the denervation studies. After surgery, the body color in the front half of the mantle on the denervated side completely disappeared (Fig. 5A), although the intact side showed unimpaired chromatic patterns. During 6 days of recovery, many of the chromatophores on the denervated side spontaneously enlarged (Fig. 5B). We analyzed the oscillatory properties of both denervated and intact areas in the test animals 6 days after the denervation procedure. The frequency distribution from the denervated areas demonstrated that the denervated chromatophores showed oscillations only below 1.5 Hz, even when many of the intact chromatophores exhibited 1–7 Hz oscillations (Fig. 5C). Therefore, these data suggest that the miniature oscillations of intact chromatophores were driven by neural activity from the CNS.

## Discussion

Although it is difficult to observe normal chromatic patterns at the single chromatophore level, we succeeded in observing chromatophore behavior in semi-intact squid displaying MDM, FES, and AD patterns (Fig. 1). After transferring intact squid to a new tank, they often displayed the MDM pattern while sitting on the bottom of the tank. This pattern may disrupt their body shape and be used for camouflage when viewed from above by predators [10], [14]. The FES pattern occasionally appeared when a squid was pinned on the chamber or the pallial nerves were cut for chromatophore denervation. In *Loligo pealei*, a male attempting to mate with a selected female often expresses the FES pattern when another male approaches [15], so this pattern may be used for intimidation. The AD pattern is clearly observed in alarmed squid [14], [16], [17]. We found that chromatophores continued to oscillate during all of these CNS-induced chromatic patterns. Intact squid that were transiently packed in a small observation chamber without anesthesia and pinning also exhibited oscillations, which indicates that the chromatophore oscillations observed in our study were not a special behavior that was observable only under our experimental conditions (see video S3). Moreover, although the direct application of a small fiber-optic light can trigger twitch contraction of chromatophores in octopus [18], we did not detect any local modifications of chromatic patterns caused by the illumination used for video recording. Our interest in this oscillatory behavior is two-fold: What does the oscillation contribute to the behavior (expansion) observed, and what can the oscillation tell us about the CNS activity behind it?

Tone matching on a background is required to achieve camouflage effects and the matching of chromatic pattern [19], [20]. What could be the mechanism for determining the expansion size of chromatophores in parallel with generation of chromatic pattern? According to brain lesion experiments in octopus, the pattern and tone are regulated by different brain regions [20]. The results from our analysis suggest that the oscillatory network in the CNS mediates a parallel processing of pattern and tone. The well-expanded chromatophores oscillated at higher frequency above 4 Hz, but the oscillation at higher frequency did not necessarily lead to the large expansion (Figs. 1Db). The spatial distribution of oscillation frequencies was chromatic pattern specific (Fig. 2). In addition, synchronization was observed among oscillations controlled by the CNS, and formation of a synchronized group was chromatic pattern dependent (Fig. 3). Our results demonstrate that the CNS drives the frequency and spatial synchronization of the miniature oscillations of chromatophores in a pattern-dependent manner. On the other hand, the chromatophores that oscillated at frequencies over 4 Hz showed a positive correlation between the expansion size and the synchronized chromatophore number (Fig. 4). Because the number of chromatophores innervated by a single motor neuron is constant [8], increasing number of synchronized chromatophores appears to be due to synchronization of the activity of different motor neurons. This result suggests that the expansion size of chromatophores oscillating at high frequency is determined by the synchrony of oscillatory behavior. The CNS activity regulating the oscillation frequency can be considered to act as a gate for induction of the large and tonic expansion of chromatophores, and to contribute to rough determination of their spatial distribution (i.e., chromatic pattern). Meanwhile, the tone of the skin, which is regulated by the exact expansion size of chromatophores [9], [10], [11], [12], may be determined by synchronous performance within the motor neural oscillations because the expansion is produced by the total activity of a set of the motor neurons innervating the specific chromatophore. It has been reported that oscillator networks are utilized for simultaneous expression or integration of different modalities of information (e.g., [21], [22]). We expect that the chromatophore control system of the squid will provide a typical and important model for understanding how the CNS integrates different modal information using an oscillator network.

A single chromatophore is controlled by different motor neurons [7], [23], thus chromatophore expansion may be elicited by ensemble activity from multiple motor neurons. Using isolated skin preparations or semi-intact preparations, electrical stimulation studies of the chromatophore nerves revealed artificial chromatophore expansions due to synchronized axon firing [6], [8], [24]. Dubas et al. [8] reported that high frequency stimuli applied to the posterior chromatophore lobe (PCL) network (in which chromatophore motor neurons are located) induced inhibitory responses of chromatophore expansion; this type of response was not induced by direct stimulation to the pallial nerve, including the chromatophore motor axons. Thus, it is possible that an inhibitory network in the CNS plays an important role in determining the motor output from the PCL. The PCL contains both motor and local neurons, and the neurons form an interactive network via electrical and chemical synapses [25]. Because it is well known that inhibitory recurrent networks are typical structures for oscillator networks in the CNS [21], it is possible that the PCL network is the original oscillator that causes the miniature oscillation of chromatophores. Dubas et al. [8] reported that chromatophore oscillations of several Hz occurred during PCL stimulation at 20 Hz, but these authors did not describe this phenomenon in detail. In our study using a semi-intact preparation, we observed oscillation behavior of chromatophores that was spontaneously produced by activity of multiple motor neurons. Denervated chromatophores showed only transient and low-frequency oscillation, as documented in previous reports [12], [20], [26], [27], [28], but they did not exhibit the miniature oscillations (Fig. 5). This result suggests that neural activities arising from the CNS are required for induction or maintenance of the oscillations. If local oscillations could be entrained into the central oscillation provided by motor neurons, the peripheral network localized on the skin might contribute to this process. Oscillatory networks that produce collective firing patterns have been found in a variety of invertebrate and vertebrate CNSs [21], [22], [29], [30], [31], and they are thought to contribute to a variety of temporal activity patterns in motor systems [32], [33], [34], [35] and assembled-activity patterns in information processing systems by using their self-organizing ability [36]. The PCL network may generate chromatic patterns in a manner similar to those of other oscillatory networks. Further analysis, mainly of the PCL, is required to reveal the nature of the oscillator system that may play an important role in chromatophore pattern expression.

## Materials and Methods

### Animals

Adult squid *Sepioteuthis lessoniana* (100–200 mm in mantle length) were obtained from fishermen fishing in Sagami Bay, Miura, Japan. Animals were maintained in a recirculating tank containing artificial seawater (Rohtomarine; Rei-Sea, Tokyo, Japan) at 19–20°C.

### Recording of chromatophore behavior of live animals

Each squid was anesthetized for 10 min in equal parts 370 mM MgCl_2_ with 10 mM HEPES (adjusted to pH 7.4 with NaOH) and seawater in the home tank. The squid was pinned dorsal side down on a 1-cm thick silicone rubber sheet (KE-103; Shin-Etsu, Tokyo, Japan) over a clear board. About 30 pins were stuck into the dorsal mantle muscle near the head through the breathing aperture and the fins along the mantle and edge. The board in which the squid was mounted was turned upside down and fixed in a container (290×200 mm) filled with normal seawater (water depth 45 mm). In this condition, the squid recovered from anesthesia for 20–25 min and spontaneously displayed chromatic patterns. More than 25 min after being transferred to the container, chromatophores on the dorsal mantle and fin were observed via a stereoscopic microscope (SMZ800; Nikon, Tokyo, Japan), and their behavior was recorded with a color CCD camera (TK4498A5; Teli, Tokyo, Japan) attached to the stereoscopic microscope. We used a cold light (MegaLight100; Schott-Nippon, Tokyo, Japan) to illuminate the dorsal mantle skin. The light intensity was 15 kLux. The image data were acquired at video rate (30 frames per second (fps)) and saved as MPEG files via an MPEG encoder (GV-MDVD2; IO Data, Kanazawa, Japan). The three main patterns (MDM, FES, and AD) analyzed in captured images (4.5×6.5 mm in view size) are described in the Results section.

### Image analysis of chromatophore behavior

To examine chromatophore behavior, we selected 3.3 s sections of the video recordings. For each period, color JPEG images were obtained at an interval of 33 ms. The size of individual black chromatophores was sequentially measured using ImageJ software (NIH, Bethesda, MD, USA) [37]. The data were analyzed using Excel software (Microsoft, Redmond, WA, USA) or Matlab software (MathWorks, Natick, MA, USA). To determine the correct frequency of the miniature oscillation, we differentiated the temporal profile of each chromatophore size to omit the influence of macroscopic expansions with lower frequency and evaluated the difference values per frame. Next we detected the rapid expansions using an amplitude threshold that was determined based on the standard deviation of the differentiated size profile. Finally, we confirmed the obtained results by examining the video to remove the influence of overestimation caused by the dynamic threshold method. To assess synchrony of the miniature oscillation among chromatophores, correlation coefficients were calculated from the differentiated size data.

### Denervation of the chromatophore system

To deprive chromatophores of neural innervation, we cut the nerves from the brain to the radial muscles of chromatophores following the procedure used by Sanders and Young [38]. After anesthetization, the pallial nerves proximal to the stellate ganglion on one side were cut with a scissor through the breathing aperture. The animals were transferred to the home tank. We observed chromatophore behavior just after the denervation and 6 days later.

## Supporting Information

Figure S1 **Temporal profile (upper) and differentiated profile (lower) of the chromatophore size for a 2 s period during the oscillation at 2 Hz ( = 500 ms period).** The differentiated profile was generated by differentiating the temporal profile of the chromatophore size. The dots on the lines were plotted at each frame (33 ms). The red areas indicate the periods of miniature expansion and the blue areas indicate the periods of miniature retraction. The chromatophore expanded within 33 ms, and then retracted in about 100 ms. The amplitude of the miniature oscillation was defined as the difference between the maximum and minimum values in each differentiated profile in the measurement period.
(TIF)

Video S1 **Dorsal mantle surface (4.7×6.5 mm) expressing chromatic pattern MDM 1 in** **Figure 3** **.** Chromatophore behavior during the 3.3 s period from 3.5 to 6.8 s after commencement of this video clip was analyzed and the data were used in Figures 2A and 3B.
(MOV)

Video S2 **Dorsal mantle surface (4.7×6.5 mm) expressing chromatic pattern MDM 2 in** **Figure 3** **.** Chromatophore behavior during the 3.3 s period from 3.5 to 6.8 s after commencement of this video clip was analyzed and the data were used in Figures 2A and 3D. Note the synchronization of miniature oscillation among some of the chromatophores in feature or background areas.
(MOV)

Video S3 **Dorsal mantle surface (9.7×13.3 mm) on intact squid which had not been anesthetized and fixed.** The squid was moving freely in a small chamber (290×200 mm). The chromatophores exhibited miniature oscillation, as in semi-intact squid impaled with pins.
(MOV)
