# Supplementary figures and images for: Chromatophore Activity during Natural Pattern Expression by the Squid Sepioteuthis lessoniana: Contributions of Miniature Oscillation
Source: PLoS One. 2011 Apr 1;6(4):e18244. doi: 10.1371/journal.pone.0018244 (PMC3069962; doi:10.1371/journal.pone.0018244)

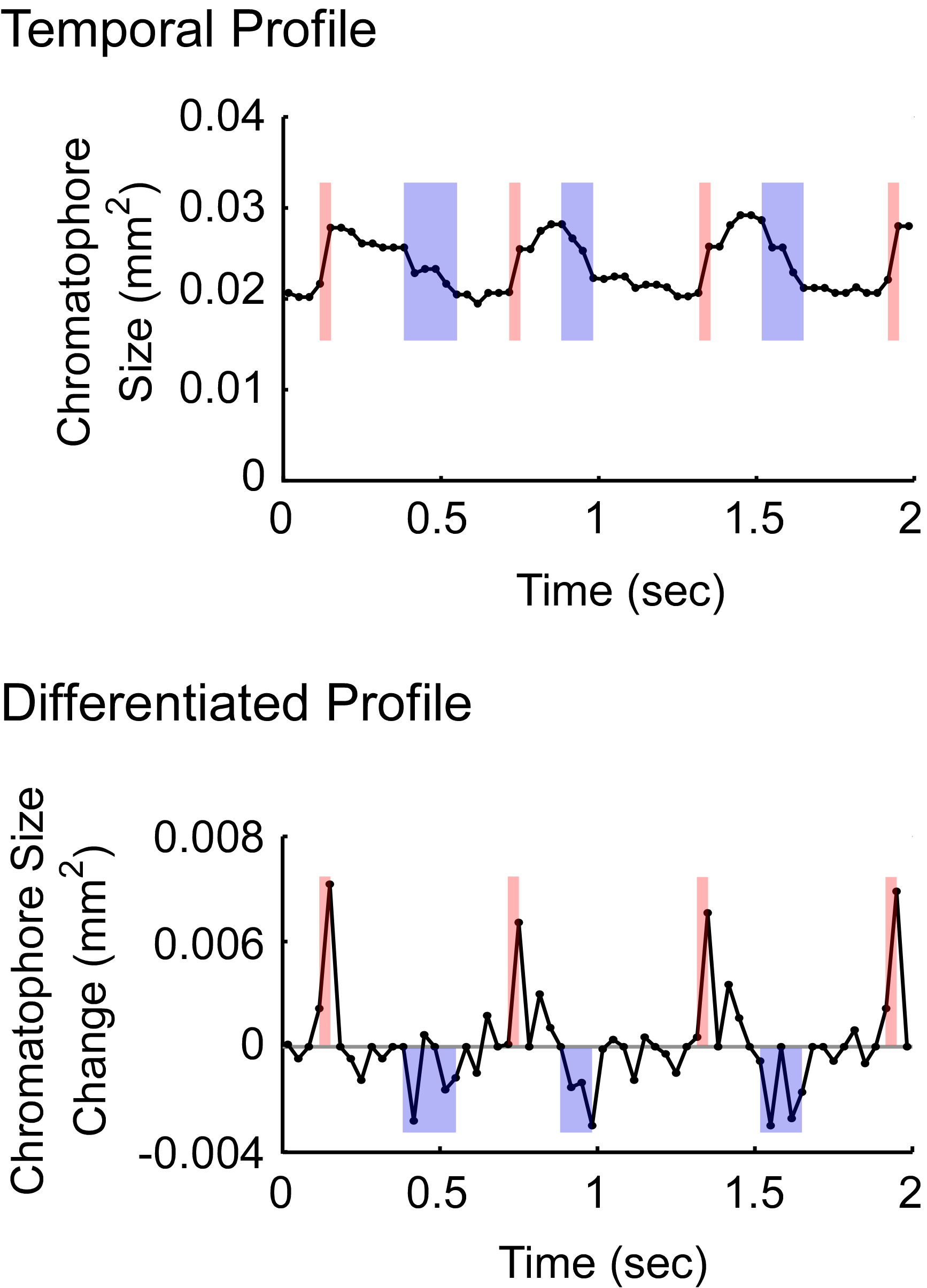

Supplement: Figure S1 — Temporal profile (upper) and differentiated profile (lower) of the chromatophore size for a 2 s period during the oscillation at 2 Hz ( = 500 ms period). The differentiated profile was generated by differentiating the temporal profile of the chromatophore size. The dots on the lines were plotted at each frame (33 ms). The red areas indicate the periods of miniature expansion and the blue areas indicate the periods of miniature retraction. The chromatophore expanded within 33 ms, and then retracted in about 100 ms. The amplitude of the miniature oscillation was defined as the difference between the maximum and minimum values in each differentiated profile in the measurement period. (TIF) [file pone.0018244.s001.tif]
